# Oral fosfomycin versus ciprofloxacin in women with *E.coli* febrile urinary tract infection, a double-blind placebo-controlled randomized controlled non-inferiority trial (FORECAST)

**DOI:** 10.1186/s12879-018-3562-2

**Published:** 2018-12-05

**Authors:** Thijs ten Doesschate, Suzan P. van Mens, Cees van Nieuwkoop, Suzanne E. Geerlings, Andy I. M. Hoepelman, Marc J. M. Bonten

**Affiliations:** 1 University Medical Centre Utrecht, University of Utrecht, Heidelberglaan 100, 3584 CX Utrecht, the Netherlands; 2 Maastricht University Medical Centre, P. Debyelaan 25, 6229 HX Maastricht, the Netherlands; 3 Haga Teaching Hospital, Els Borst-Eilersplein 275, 2545 AA The Hague, the Netherlands; 4 University of Amsterdam, Meibergdreef 9, 1105 AZ Amsterdam, the Netherlands

**Keywords:** Fosfomycin-trometamol, Urinary tract infection, *Scherichia coli*, Antibiotic treatment, Randomized clinical trial

## Abstract

**Background:**

Febrile Urinary Tract Infection (FUTI) is frequently treated initially with intravenous antibiotics, followed by oral antibiotics guided by clinical response and bacterial susceptibility patterns. Due to increasing infection rates with multiresistant Enterobacteriaceae, antibiotic options for stepdown treatment decline and patients more frequently require continued intravenous antibiotic treatment for FUTI. Fosfomycin is an antibiotic with high bactericidal activity against *Escherichia coli* and current resistance rates are low in most countries. Oral Fosfomycin-Trometamol 3000 mg (FT) reaches appropriate antibiotic concentrations in urine and blood and is considered safe. As such, it is a potential alternative for stepdown treatment.

**Methods:**

The FORECAST study (Fosfomycin Randomized controlled trial for *E.coli* urinary tract infections as Alternative Stepdown Treatment) is a randomized, double-blind, double-dummy, non-inferiority trial in which 240 patients will be randomly allocated to a stepdown treatment with FT or ciprofloxacin (standard of care) for FUTI, caused by *Escherichia coli* with in vitro susceptibility to both antibiotics*.* The study population consists of consenting female patients (≥18 years) with community acquired *E. coli* FUTI. After intravenous antibiotic treatment during at least 48 (but less than 120) hours, and if eligibility criteria for iv-oral switch are met, patients receive either FT (3 g every 24 h) or ciprofloxacin (500 mg every 12 h) for a total antibiotic duration of 10 days. The primary endpoint is clinical cure (resolution of symptoms) 6–10 days post-treatment. Secondary endpoints are microbiological cure 6–10 days post-treatment, clinical cure, mortality, ICU admittance, relapse, reinfection, readmission, additional antibiotic use for UTI, early study discontinuation, adverse events, days of hospitalization and days of absenteeism within 30–35 days post-treatment. The sample size is based on achieving non-inferiority on the primary endpoint, applying a non-inferiority margin of 10%, a two-sided *p*-value of < 0.05 and a power of 80%.

**Discussion:**

The study aims to demonstrate non-inferiority of oral fosfomycin, compared to oral ciprofloxacin, in the stepdown treatment of *E. coli* FUTI.

**Trial registration:**

Registered at the Nederlands trial register (Dutch trial register) on 4-10-2017. Trial registration number: NTR6449. Secondary ID (national authority): NL60186.041.17.

## Background

Urinary Tract Infections (UTI) are the most common bacterial infections requiring antibiotic treatment in the western world [1]. Complicated urinary tract infections (cUTI) are defined upon the presence of systemic symptoms or upon the susceptibility of the host for a complicated course [2]. Systemic symptoms are often fever, febrile UTI (FUTI), or other symptoms, reflecting the presence of tissue infection such as pyelonephritis, prostatitis or the urosepsis syndrome [2]. Guidelines recommend to treat FUTI with a 7–14 day course of antibiotics [2, 3] and in the majority of cases empiric intravenous antibiotics will be followed by oral stepdown therapy. The choice of stepdown treatment should be targeted to the susceptibility pattern of the causal uropathogen. Unfortunately, antibiotic options for stepdown treatment are becoming limited as the result of increasing antibiotic resistance [2].

*Escherichia coli* (*E.coli*) is the causative organism in around 70–75% of FUTI [4, 5]. In 2017, resistance rates of *E. coli* isolates of patients admitted to Dutch hospitals were 14% for ciprofloxacin (CPX), 23% for trimethoprim-sulfamethoxazole and 36% for amoxicillin-clavulanic acid with higher resistance rates in patients from urology departments [6]. Though this leaves fluoroquinolones still as one of the oral antibiotics to treat FUTI, one of the goals of antibiotic stewardship is to reduce fluoroquinolone use because of its selective properties for antibiotic resistance [2]. In the Netherlands, about 2–5% of patients hospitalized for community-acquired FUTI cannot be treated with oral antibiotics due to antibiotic resistance of the causal uropathogen [7]. This implies the need of prolonged intravenous antibiotic treatment, prolonged hospitalization, increased healthcare costs and higher risks of forthcoming complications.

Fosfomycin is an alternative antibiotic treatment for the stepdown treatment of FUTI [8, 9]. Fosfomycin is a phosphoenolpyruvate analogue and orally available as Fosfomycin-Trometamol (FT). It has been used extensively for uncomplicated cystitis in women, has a good safety profile [10] and possesses a suitable pharmacokinetic profile in healthy volunteers.

In the FORECAST trial (FOsfomycin Randomized controlled trial for *E.coli* urinary tract infections as Alternative Stepdown Treatment), we will investigate the efficacy of FT when used as stepdown therapy of FUTI in women.

## Methods

The objective of this randomized controlled double-blind, double-dummy, non-inferiority, multicentre, investigator-initiated trial is to determine whether oral FT is non-inferior to oral CPX in the step-down treatment of *E.coli* FUTI in women for achieving clinical cure. The study will be performed in 15 Dutch hospitals; 4 academic centers and 11 large teaching hospitals.

### Participants

#### Inclusion criteria

Competent women (≥18 years), that are hospitalized for the presumed diagnosis FUTI and receive appropriate empirical intravenous antibiotics for ≥48 - ≤120 h, who are judged to be eligible for an intravenous-oral switch by the attending physician, according to the Dutch guideline, that recommends to switch therapy until the patient has been afebrile for 24–48 h and symptoms have improved [2]. Urine (≥10^4^ CFU/ml) or blood culture must reveal *E.coli*, susceptible to both CPX and fosfomycin. A patient is not eligible if non-E.*coli*-type Enterobacteriaceae are present in urine culture (≥10^3^ CFU/ml) or blood culture.

FUTI is defined as UTI with at least one forthcoming systemic symptom or sign and one local symptom at presentation. Local symptoms are dysuria, urinary urgency, urinary frequency, suprapubic/pelvic discomfort, macroscopic hematuria, new urinary incontinence or worsening of pre-existing incontinence, lower abdominal pain, low back pain, flank pain, costo-vertebral angle pain or tenderness on physical examination. Systemic symptoms or signs are fever (≥38.0 C ͦ) or low temperature (< 36 C ͦ), rigors, delirium, hemodynamic instability as a result of sepsis requiring intravenous fluids or an increase in CRP (≥30 mg/L) or leucocytes (≥12*10^9^/L). Local symptoms are not required in case the urine and blood culture yield are positive for a phenotypically identical matched E.coli and UTI is the presumed source of infection according to the treating physician.

Adequate empirical intravenous treatment may consist of amoxicillin+/−clavulanic acid, 2nd or 3rd generation cephalosporin, aminoglycoside, carbapenem, fluoroquinolones, trimethoprim-sulfamethoxazole or a combination and in vitro susceptibility of the causative *E.coli* to at least one of the used agents.

#### Exclusion criteria

Pregnancy or breastfeeding, glomerular filtration rate below 30 ml/min/1,73 m3 or renal replacement therapy, concomitant systemic antibacterial treatment, ascertained or presumptive hypersensitivity to (compounds of) the study products, participation in any trial with an investigational product involved in the 30 days before the screening visit, specific patient groups: patients with renal transplant, polycystic kidney disease, neutropenia (< 500 /μl), paraplegia, suspicion/presence of renal abscess, suspicion of septic metastatic foci/endocarditis, urostomy, ileal loops, long-term urinary catheter (placed ≥24 h before admission), e.g., double-J catheter, nephrostomy catheter, suprapubic catheter, specific contra-indications for CPX or fosfomycin: concurrent use of tizanidin, clozapin or theophylline, a history of tendon disease/disorder related to quinolone treatment, patients with known risk factors for prolongation of the QT interval, glucose-6-phosphate dehydrogenase deficiency, inadequate understanding of the study risks or its requirements or unwilling to plan a follow-up visit and every other laboratory result, clinical condition, disease or treatment that, in investigator’s opinion, make the subject non suitable for the study. Patients with partial obstruction of the urinary tract, ureteral stones or intermittent catheterization will not be excluded.

### Setting

Potential subjects for inclusion will be identified through daily screening for *E. coli* in urine and blood cultures in the clinical microbiology laboratories of participating centres. The study investigator will assess potential subjects for eligibility with consultation of the attending physician. After identification, eligible patients will be informed and, after obtaining consent, will be randomized.

### Randomization

The data manager of the University Medical Centre Utrecht constructed a randomization list, that connects the unique medication code with the active substance (1:1). Only the data manager and the pharmacist on duty, responsible for the deblinding process in case of emergency, have access to the randomization code. Constrained randomization will take place with permuted blocks, that contain a pre-specified number of treatment assignments in a random order. As we expect to include more than 200 participants in a two-arm trial, the probability of a significant imbalance is negligible [11]. To prevent imbalances between both study arms stratification will be performed per study centre, as each study centre handles their own local guideline for the empirical treatment of FUTI.

### Treatment

Participants will be randomized for an intravenous-oral antibiotic switch to either FT 3000 mg every 24 h or CPX 500 mg every 12 h [1]. CPX is chosen for the control group, because most evidence exists for CPX as an oral treatment of FUTI [12, 13]. A double-dummy design is chosen as it is impossible to equalize fosfomycin granules to any form of CPX. An identical placebo will be used for both active substances. All medicines will be manufactured according to Good Manufacturing Principles by the pharmaceutical company Basic Pharma BV, the Netherlands. The duration of total antibiotic therapy is 10 days for all participants, of which 2–5 days empirical intravenous antibiotics and the remaining 5–8 days stepdown therapy with FT or CPX.

FT will be dosed 3 g every 24 h, which is based on a review of the scientific data and expert opinion on the treatment of FUTI and the pharmacokinetics of FT that was available at the moment the study was designed (May 2017). The use of FT every 24 h for more than a single dose was tolerated well in retrospective studies and according to personal off-label use [14]. We do not expect negative effects of accumulation as fosfomycin is registered for intravenous use in dosages as high as 24 g per 24 h [15]. A medication diary needs to be filled by the participant in order to improve medication adherence. All patients receive oral and written information on the trial, including possible risks of participating. Additionally, patients receive a medication folder with instructions for use, contra-indications, interactions and adverse events of both FT and CPX (probable, possible and seldom adverse events). For questions or concerns, participants could contact the local or principle investigator or an independent expert physician.

### Data collection

In each hospital, authorized and qualified clinical investigators and research nurses will obtain informed consent for participation. Demographic, clinical and microbiological data will be collected and stored for at least 15 years. Data will be collected from questionnaires and supplemented with data from the electronic medical record, see Table 1.

**Table 1:** Enrolment, interventions and assessments in the FORECAST study

	Patient identification	Before randomization	Study treatment (5–8 days)	6–10 days post-end of treatment	30–35 days post-end of treatment
Enrolment:					
Screening for eligibility	x				
Entry criteria		x			
Informed consent		x			
Interventions:					
Venapunction^a^		x			
Urine/blood culture	x			x (urine)	
Study treatment			x		
Assessments:					
Electronic patient file		x		x	x
Patient questionnaire		x		x	x
Hand in study diary and residual study medicines				x	

^a^in case of doubt about the following exclusion criteria: pregnancy, neutropenia or renal insufficiency

All participating microbiological and clinical chemistry laboratories are accredited by the Dutch foundation for the promotion of the quality and the accreditation of laboratories in health care (ISO). Susceptibility to fosfomycin and CPX is tested using validated local susceptibility methods, following the European Committee on Antimicrobial Susceptibility (EUCAST) recommendations. The clinical (*E. coli*) isolates will be stored for additional susceptibility measurements, i.e. agar dilution for fosfomycin susceptibility, and molecular analysis.

The primary and secondary endpoints will be collected 6–10 days post-end-of-treatment during a visit and 30–35 days post-end-of-treatment by telephone. Data will be entered into the Case Report Form (CRF) pseudo-anonymously in each participating centre using an unique participant code. The data will be processed by the data management program ‘ResearchOnline’. All steps of the data process will be stored to be able to check validity and plausibility.

### Endpoints

The primary endpoint is clinical cure (resolution of symptoms) 6–10 days post end-of-treatment. Definitions and criteria of primary and secondary endpoints are described in Table 2.

**Table 2:** Endpoints, provided with definitions and a time frame

Endpoint	Definition	Time frame
Clinical cure	Alive with reduction of all initial local and systemic FUTI related symptoms and without additional systemic antibiotic therapy for UTI (except antibiotic prophylaxis)	6–10 days post-end of-treatment (PET) + 30–35 days PET
Microbiological cure	Negative urine culture for E.coli (< 10^3^ CFU/ml), phenotypically identifiable to the initial culture (assessment by microbiologist) ^a^	6–10 days PET
Acquired resistance	Resistance to ciprofloxacin, fosfomycin or new ESBL-producing bacteria in phenotypically identical strain	6–10 days PET
Mortality	-Mortality for any reason	Within 30–35 days PET
	-Mortality related to UTI or study medicines	
ICU admission	-ICU admission for any reason	Within 30–35 days PET
	-ICU admission related to UTI or study medicines	
Readmission	-Readmission for any reason	Within 30–35 days PET
	-Readmission related to UTI or study medicines	
Relapse	Development of new symptoms of UTI after previous clinical and microbiological cure with a phenotypically identical strain as isolated during the initial blood or urine (≥10^3^) cultures.	Within 30–35 days PET
Reinfection	Same definition as relapse, but with phenotypically different strains isolated in cultures (urine, ≥10^3^)	Within 30–35 days PET
Additional antibiotic use	Additional systemic antibiotic therapy for UTI (except antibiotic prophylaxis)	Within 30–35 days PET
Length of hospital stay	-Total days of hospital stay	Within 30–35 days PET
	-Total days of ICU stay	
Days of absenteeism	Converted to full work days:	Within 30–35 PET
	-Paid work	
	-Voluntary work	
Adverse events	Possible or probable related to study protocol	Within 30–35 days PET
Early study medicine discontinuation	Early study medicine discontinuation:	–
	-because of intolerance/adverse events	
	-because of clinical failure	
	-because of resolution of symptoms	

^a^Other strains in urine culture will be reported, but do not fall within this definition

### Ethics

This study has been set up in accordance with the principles of the Declaration of Helsinki and Good Clinical Practice guidelines. The medical ethics committee of the University Medical Centre Utrecht approved the study protocol, followed by the Institutional Scientific Boards of each participating centre. Written informed consent is obtained prior to randomization. Structural protocol modifications, including amendments, will be communicated as soon as possible to the medical ethics committee, the trial register, and all involved investigators and participants.

### Statistical analysis

#### Sample size calculation

We determined the sample size based on an assumed cure rate of 92.5% for both groups and a margin of inferiority of 10%, consistent with assumptions in similar UTI trials [5, 12, 13, 16]. Assuming that a difference of 10.0 percentage points or less is irrelevant and with the two-sided alpha set at 0.05, the sample size needed in the two groups is 109. The study has a 80% power to reject the null hypothesis that the clinical cure rate for the study arm (FT) is 10% lower than the cure rate for the standard of care (CPX). Equivalently, the likelihood is 80% that the two-sided 95.0% confidence interval for the difference in clinical cure rates will exclude a 10% difference in favour of standard care. In order to evaluate 109 patients in both treatment arms, taking into account 10% of lost participants for various reasons, 240 subjects will be enrolled in total.

#### Intention to treat and per-protocol analysis

Participants are evaluable if they are randomized and received at least one dose of the study drug, and all will be included in the intention to treat analysis. Analysis of all endpoints will be performed according to the intention-to-treat principle, on which data non-inferiority is based. A per-protocol analysis will be performed for the primary endpoint and the secondary endpoint ‘microbiological cure’ for patients that completed at least 80% of study medicines.

#### Statistical methods

For the primary endpoint, the difference between the study arm and standard of care (*p* < 0.05) will be calculated with a two-sided Z-score for proportions. Secondary outcomes will be analysed using the following tests: two-tailed Z-score for proportions (p < 0.05), t-test, chi-square, logistic regression, when appropriate.

Linear and logistic regression will be used to identify associations between patient, disease and treatment characteristics) with both study arms with regard to the primary and secondary endpoints. Clinical cure 6–10 and 30–35 days post-treatment in specific subgroups will be investigated in exploratory analyses, based on host or disease characteristics, e.g. age, BMI, Charlson index, use of immunosuppressive drugs, presence of host factors, presence of a short term indwelling catheter, creatinine clearance, diabetes mellitus, days of empirical intravenous antibiotics, class of empirical intravenous antibiotics, and concomitant bacteraemia.

#### Missing data

Attempts will be made to complete the data from all enrolled participants. Missing data will be tracked or retrieved by the coordinating team after consultation with the local team. Missing information will be extracted directly from the electronic patient file without disclosing patient identifiers. Multiple imputation will be used for missing data.

#### Safety

Our entry criteria are designated to reflect daily practice with regard to the safety of the participants. Candidates should be diagnosed with FUTI as the primary reason for hospitalization. All identified uropathogens must be *E. coli* susceptible to both fosfomycin and CPX. Patients should be treated with appropriate intravenous antibiotics for at least 48 h. The attending physician determines the eligibility for the intravenous-to-oral switch. Patients that require more than 5 days of intravenous antibiotics will be excluded as this is considered a complicated course. Our goal is to investigate a representative population by including as many patients as possible, and only exclude extremely vulnerable patients (e.g. non-competent patients, renal transplant recipients, pregnant women), patients that require an alternate antibiotic strategy (e.g. renal abscess, endocarditis) or patients for which other endpoints should be used to estimate efficacy (e.g. men). Adverse events should be actively reported in the medication diary by participants in order to measure tolerability. Furthermore, these will be checked actively with questionnaires at 6–10 days and 30–35 days post-end of treatment. De-blinding is possible 24/7 h in case severe harm could be potentially prevented, and after consultation of the coordinating investigator. Serious adverse events and Suspected unexpected serious adverse reactions (SUSARs) will be reported during follow-up. All participants are automatically insured by a subject and liability insurance.

The study will be monitored by qualified monitors from the University Medical Centre Utrecht. For each participating centre the following procedures will be checked after the first 5 inclusions and thereupon annually: at least 10% of signed Informed Consent forms, in- and exclusion criteria, defined variables including the primary endpoint, source data verification, missed SAE’s, or SUSAR’s.

An interim analysis will be performed by the Data and Safety Monitoring Board (DSMB) after inclusion of 50 and 100 participants. De-blinded data will be delivered by an independent statistician to the DSMB, which will function as an advisory board, i.e. it will provide non-binding advice to the principal investigator. After inclusion of 50 participants an interim analysis will be performed regarding adverse events, medication adherence and early study medicine discontinuation as a result of adverse events/intolerance. Based on these results, the DSMB could recommend to adjust the dosage of FT. After inclusion of 100 participants, an interim analysis will be performed regarding Serious Adverse Events (SAE’s), early study withdrawal and the primary endpoint. Based on these results, the DSMB could recommend to stop the study, in case the interim analysis shows a probability of finding non-inferiority at the final analysis of less than 5% based on the conditional power (futility) [17] or an unexpected high rate of possible related SAEs or early study withdrawals, in particular in the FT arm (safety).

## Discussion

As a result of emerging antibiotic resistance among Enterobacteriaceae the options for oral antibiotic stepdown treatment of FUTI are becoming limited. Whereas new intravenous antibiotics are being developed and registered, little interest has been put in the development of oral applications in the past decade [4, 16, 18]. As a consequence, patients with FUTI caused by (or carrying) a multiresistant Enterobacteriaceae usually require full intravenous antibiotic regimens with a higher risk on subsequent complications, psychological burden and increased health care costs, partly due to the forthcoming extended length of hospital stay [19].

This has led to renewed interest in FT, a relatively infrequent used antibiotic, invented in 1969 [20]. FT could be a suitable choice for targeted stepdown treatment of *E.coli* FUTI. FT was non-inferior in comparison to carbapenems in observational studies considering cUTIs [8, 9]. This investigator-initiated randomized controlled trial aims to evaluate FT as stepdown treatment for *E.coli* FUTI in women.

The eligibility criteria of this study were defined such that results will be generalizable to the population being treated for FUTI in clinical practice. Consequently, patients with FUTI with a positive urine or blood culture revealing E.*coli* will be included if both local and systemic UTI signs or symptoms are present and the patient is treated with adequate intravenous antibiotics with the presumptive focus of infection being the urinary tract. The presence of fever is not required for enrolment, as we aimed to include certain vulnerable patient populations that sometimes do not develop fever (i.e. elderly, immunocompromised patients) during sepsis. In order to better fit our domain, minor changes were made in eligibility criteria after we recruited 15 participants [1]: As a systemic sign, we included an increase in CRP (≥30 mg/L) or leucocytes (≥12*109/L) [2]. Next, local symptoms are not required in case the urine and blood culture are positive for a phenotypically matched E.*coli* and UTI is the presumed source of infection according to the treating physician [3]. Short term urinary catheters, if placed at least within 24 h before admission, will be allowed, e.g. double-J catheter, nephrostomy catheter, suprapubic catheter [4]. Finally, a patient is not eligible if any non-E.coli-type Enterobacteriaceae are present in urine (≥10^3^ CFU/ml) or blood culture.

As primary endpoint, clinical cure 6 to 10 days post-end of treatment was chosen, with, next to safety endpoints, microbiological cure at 6–10 days and clinical cure at 30–35 days post-end of treatment as secondary endpoints. For future perspectives, cost-effectiveness variables will be collected, i.e. length of hospital and intensive care stay and days of absenteeism, in order to perform a model-based cost-effectiveness analysis between oral FT as stepdown treatment versus a full intravenous antibiotic course (e.g. with carbapenems) for the treatment of multiresistant E.*coli* FUTI.

There were several aspects related to the spectrum of FT that guided the study design. First, it is not rational to use FT for the empirical treatment of FUTI. The pharmacokinetic-pharmacodynamics profile is presumably not suitable to treat FUTI empirically. One gift of 3 g (oral) FT results in a peak fosfomycin urinary concentration of 1.600 mg/L and concentrations above the Minimal Inhibitory Concentration (MIC) of most *E.coli* isolates (below 2-4 mg/L including Extended Spectrum Beta-Lactamase-ESBL) for about 48 h [21–23]. The same gift leads to a peak serum concentration of around 20 mg/L, declining below 4 mg/L within 8–12 h [21, 24]. Second, in our opinion, FT is investigated for its use as targeted stepdown treatment of *E.coli* pathogens only, as *Klebsiella* and *Proteus* spp. are less susceptible to fosfomycin with proportions of resistance to fosfomycin of 31 and 16%, respectively, in the Netherlands [25]. The prevalence of resistance to fosfomycin among *E.coli* isolates from urine samples has been stable for years (+ − 1%) in the Netherlands [25], and is even low among ESBL-producing Enterobacteriaceae [26]. Finally, determination of fosfomycin susceptibility in daily practice seems more reliable for *E.coli* than for non-*E.coli* Enterobacteriaceae [27]. Third, although FT has been used in men to treat prostatitis and FUTI, we decided not to include men as they require longer antibiotic treatment and because treatment failure expresses itself as relapses for an extended period of time [12]. Stratification for gender has been considered, but would, within the limits of perceived feasibility, reduce statistical power to draw meaningful conclusions about non-inferiority. Finally, a study that was published recently evaluated the tolerability and pharmacokinetics of using FT every 48 h for three doses versus every 24 h for 7 doses [24]. The ‘every 24 hours’ arm was associated with significantly less days of being free of diarrhoea, however no subject discontinued FT due to adverse events. The study was not placebo-controlled, and was performed in healthy volunteers, therefore it is unknown how this reflects on the participants in this study. For safety reasons, adverse events and adherence will be evaluated during the interim analyses after 50 participants. Currently, the FOREST study (http://www.clinicaltrial.gov, NCT02142751) compares the efficacy of intravenous fosfomycin to meropenem for bacteraemic ESBL-*E.coli* UTI. In this study, intravenous fosfomycin is followed by stepdown therapy with 3 g FT every 48 h [18].

In summary, the FORECAST study will determine the efficacy of fosfomycin-trometamol, compared to CPX, in the stepdown treatment of *E.coli* FUTI in women.

## Abbreviations

CPX: Ciprofloxacin
CRF: Case Report Form
DSMB: Data and Safety Monitoring Board
E.coli: Escherichia coli
ESBL: Extended Spectrum Beta-Lactamase
EUCAST: European Committee on Antimicrobial Susceptibility
FORECAST: FOsfomycin Randomized controlled trial for E.coli Complicated urinary tract infections as Alternative Stepdown Treatment
FT: Fosfomycin-Trometamol
FUTI: Urinary Tract Infection with systemic symptoms or signs
MIC: Minimal Inhibitory Concentration
SAE: Serious Adverse Event
SUSAR: Suspected Unexpected Serious Adverse Reactions
UTI: Urinary Tract Infection
